# *CYP2D6* Genotype and Pharmacovigilance Impact on Autism Spectrum Disorder: A Naturalistic Study with Extreme Phenotype Analysis

**DOI:** 10.3390/ph16070954

**Published:** 2023-07-03

**Authors:** Pura Ballester, Cristina Espadas, Susana Almenara, Jordi Barrachina, Javier Muriel, Enrique Ramos, Natalia Toral, César Belda, Ana M. Peiró

**Affiliations:** 1 Pharmacology Department, Pharmacy Degree, San Antonio Catholic University, 30107 Murcia, Spain; 2 Bioengineering Institute, Pediatrics and Organic Chemistry Department, Miguel Hernández University of Elche (UMH), 03202 Alicante, Spain; cristina.espadas@goumh.umh.es (C.E.); apeiro@umh.es (A.M.P.); 3 Neuropharmacology on Pain Treatment and Neurodevelopmental Disorders, Dr. Balmis General University Hospital, Alicante Institute for Health and Biomedical Research (ISABIAL), 03010 Alicante, Spain; susanaalmenara@gmail.com (S.A.); jordibernabeu15@gmail.com (J.B.);; 4 Clinical Pharmacology Unit, Alicante General University Hospital, 03010 Alicante, Spain; 5 Clinical Pharmacology, Pediatrics and Organic Chemistry Department, Miguel Hernández University of Elche (UMH), 03202 Alicante, Spain; 6 San Rafael Center—San Francisco De Borja Foundation, Residential Facility, 03559 Alicante, Spain; 7 Infanta Leonor Center, Autism Parents Association Valencian Community Autism Association (APACV), 03010 Alicante, Spain

**Keywords:** autism, *CYP2D6*, pharmacogenetics, adverse events, polypharmacy

## Abstract

The long-term use of psychopharmacology medications in autism spectrum disorder (ASD) hitherto remains controversial due to a lack of evidence about safety and tolerability. In this regard, genotyping the metabolizing enzyme cytochrome P450 *(CYP) 2D6*, especially its extreme phenotypes, could help to prevent drug-related adverse reactions or adverse events (AEs). There are several medications warranting *CYP2D6* screening that are consumed by people with ASD, such as risperidone and aripiprazole to name a few. A naturalistic observational study was carried out in participants with ASD to analyze the influence of the *CYP2D6* phenotype in drug tolerability using a local pharmacovigilance system created for this study. In this case, AEs were identified from participants’ electronic health records (EHRs) and paper registries. Other variables were collected: socio-demographic information, comorbidities, and psychopharmacology prescriptions (polypharmacy defined as ≥4 simultaneous prescriptions) and doses. The genetic analysis included allelic discrimination (CYP2D6*1, *2, *3, *4, *5, *6, *10, *17, and *41) and copy number variations. All of these were used to determine theoretical phenotypes of the metabolic profiles: poor (PM); intermediate (IM); normal (NM); and ultra-rapid (UM). Sex differences were analyzed. A total of 71 participants (30 ± 10 years old, 82% male, 45% *CYP2D6* NM phenotype (32 participants)) with a median of 3 (IQR 2–4) comorbidities per person, mainly urinary incontinence (32%) and constipation (22%), were included. *CYP2D6* UM showed the highest rate of polypharmacy, whilst, IM participants had the highest rates of neurological and psychiatric AEs, even worse if a *CYP2D6* inhibitor drug was prescribed simultaneously. *CYP2D6* pharmacogenomics and the monitoring of new antipsychotic prescriptions may make a difference in medication safety in adults with ASD. Particularly in those with psychopharmacology polymedication, it can help with AE avoidance and understanding.

## 1. Introduction

Pharmacological treatment in individuals with autism spectrum disorder (ASD) is complex due to the scarce number of treatments approved by drug agencies and the wide variability in comorbid conditions (hyperactivity, depression, or anxiety disorders) [1,2]. The lack of clinical guidelines to treat these comorbidities is accompanied by high rates of polypharmacy and a wide range of drug adverse events (AEs) [3]. Nowadays, for providers it is a challenge to distinguish between AEs and comorbidities [4]. The conditions that surround an ASD diagnosis tend to increase in patients by anywhere from 7 to 12 times the chance of a new medication prescription [5]. Further research is needed to elucidate the prescribing cascade and deprescribing strategies in ASD, bearing in mind the considerable difficulty of standardizing ‘patient monitoring’ and long-term follow-up with deprescription programs.

In addition, the long-term use of psychopharmacology treatments in ASD, especially when multiple and simultaneous medications are taken, remains controversial, and few efforts have been made to evaluate safety and tolerability [6,7]. Polymedication leads to a negative correlation with overall good health status, remarkably, in young individuals. Against this backdrop of uncertainty, the pharmacogenomics of cytochrome P450 (CYP) 2D6 could help to develop individualized therapy before prescription in ASD. The *CYP2D6* enzyme is involved in the metabolism of most antipsychotic and antidepressant drugs [8]. Both drug groups represent more than half the medications usually prescribed in ASD [9], as risperidone is extensively metabolized to the active metabolite 9-hydroxyrisperidone [10]. Some *CYP2D6* polymorphisms are associated with enzymes’ normal, absent, increased, or decreased activity, which can comprise drug concentration in plasma [11,12]. These variants can affect drugs’ pharmacological actions, effectiveness, or tolerability [13]. Extrapyramidal symptoms, sedation, hyperprolactinemia, and weight gain may appear as drug side-effects at a higher rate [14]. In addition, individuals with an ultra-rapid metabolizer (UM, increased enzyme activity) phenotype are expected to exhibit faster drug clearance rates, resulting in unresponsiveness to treatment [15]. Conversely, poor metabolizers (PMs) may require a smaller medication dose [16] and experience higher frequencies of AEs [17] potentially related to drug accumulation [18]. It has been defined that sex impacts the rates of AEs found [19]. PMs tend to have either lower levels of active metabolites [20] and, therefore, reduced efficacy when it depends on drug metabolite action [21] or a lesser efficacy for standard doses of medications activated by *CYP2D6* metabolism [22].

The main objective of this study is to evaluate the prevalence of comorbidities and AEs in ASD and to study their relationship with *CYP2D6*-metabolizing phenotype, all with the view of pursuing the optimization of all simultaneous medications taken by the participants. To illustrate the results, for some a case report is also provided.

## 2. Results

A total of 145 people diagnosed with ASD were prescreened, with 124 subjects being candidates according to the inclusion criteria; 21 participants were no longer candidates since their medications were not metabolized by the cytochrome analyzed or because blood samples were not possible for DNA extraction. The sample size was then reduced to 83 adults analyzed, with 12 (14%) losses during follow-up [2]. So, due to the impossibility of obtaining pharmacological data or a large enough blood sample to carry out the genetic study, the sample size was reduced to 71 adults.

### 2.1. Demographic and Clinical Data

Our study involved the comprehension of comorbidities and AEs in an adult population with ASD considering CYPD26-metabolizing phenotype. Table 1 and Table 2 record demographic and comorbidities data.

**Figure 1:**
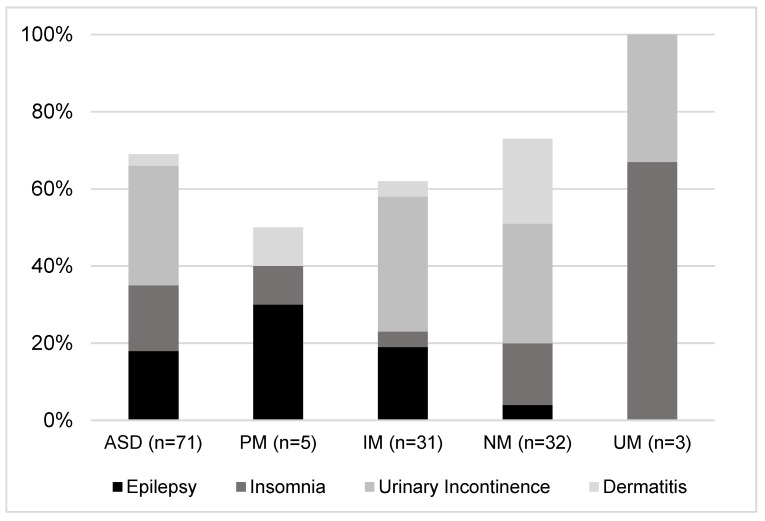
Sample split by *CYP2D6* phenotype (poor metabolizers (PMs), intermediate metabolizers (IMs), normal metabolizers (NMs) or ultra-rapid metabolizers (UMs)), describing the most prevalent comorbidities per group.

Our population was mainly adult males (30 ± 10 years, 86%), with a total of two hundred and twenty-two comorbidities (median of 3 (IQR 2–5) per person) grouped into ninety different diagnosis that encompassed both physical problems and psychological disorders. The distributions of the main comorbidities observed in each group are displayed in Figure 1. Nervous and psychiatric comorbidities stood out in the phenotypes, with ID (20%), epilepsy (18%), insomnia (17%), psychotic agitation (17%), and anxiety (8%) as the highest representatives.

In this study, more than half of the population (58%) was classified as overweight (mean BMI, 27 ± 6 Kg/m^2^), but only 17% had a diagnosis in their clinical records, and any sleeping problems were registered in participants’ EHRs. In addition, according to the inclusion criteria, all subject participants in the study had an ID diagnosis; however, only 20% had this diagnosis in their EHRs. Therefore, these three comorbidities were under-reported.

### 2.2. CYP2D6 Phenotype Influence

**Figure 2:**
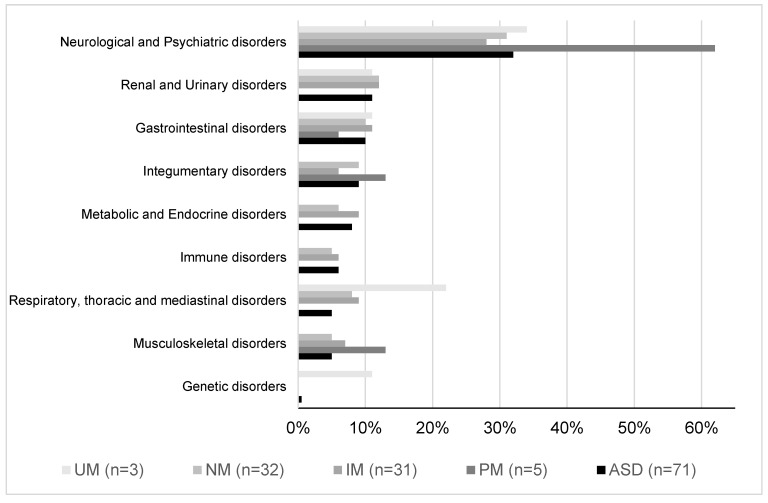
AEs or disorders classified by *CYP2D6* phenotype (poor metabolizers (PMs), intermediate metabolizers (IMs), normal metabolizers (NMs), or ultra-rapid metabolizers (UMs)) in the population.

The next step was to perform the *CYP2D6* enzyme genotyping, which obtained the following proportions amongst the study population: 4% UM, 7% PM, 44% IM, and 45% NM. Knowing the phenotype served to group the population and study the rates of different phenomena, such as comorbidities or medication doses. Comorbidity distribution (n = 71, median 3, IQR 2–5) was significantly different when classified by *CYP2D6* phenotype. PM subjects presented mainly comorbidities related to the nervous system and psychiatric disorders (62%) and a few related to musculoskeletal, skin, or blood disorders. Those who displayed an IM phenotype evidenced, with less frequency than PMs, nervous and psychiatric disorders (28%). However, also worthy of mention were the comorbidities related to the renal and gastrointestinal systems (12 and 11%, respectively). NMs showed similar results to IMs for the nervous system and psychiatric, renal, and gastrointestinal comorbidities (31%, 12%, and 9%, respectively). Finally, UM subjects showed a median of three (IQR 1–5) comorbidities per person. Three of these corresponded to nervous system and psychiatric disorders. This group also presented one comorbidity related to genetic disorders not observed in any other phenotype (see Figure 1 and Figure 2).

### 2.3. Pharmacology Variables

Table 3 records a total of 55 different active ingredients prescribed that are classified according to mechanism of action. Attending the whole population, an amount of three hundred and thirteen prescriptions was recorded, with a mean of four (IQR, 3–6) per subject. This represents a prevalence of 56% polypharmacy, mainly due to antipsychotics (50%), followed by anticonvulsant (23%), anxiolytic (15%), and antidepressant (12%) drugs.

In addition, when analyzing the risk of presenting AEs considering participants’ sex, age, BMI, comorbidities, metabolic phenotype, and number of ongoing medications (antipsychotics, antidepressants, anxiolytics, and anticonvulsants), the results showed that adding a new antipsychotic to a participant’s prescription significantly increased the presentation of adverse events by 33% if a participant already had a *CYP2D6* inhibitor (*p* = 0.000 < 0.05, R square = 0.322).

### 2.4. CYP2D6 Phenotype Influence

A contingency table analysis showed a statistically significant association (*p* < 0.0001) between the dose administered and the metabolizing phenotype of patients who had experienced AEs (see Table 4). Thus, drugs at the DDR were mostly administered to the EM population, while PMs were more likely to have drug doses outside the usual DDR, and UMs were less likely to have ^SUP^DDR. On the other hand, *CYP2D6* substrates were mainly drugs at the DDR, while those administered at INFDDR were both substrates and inhibitors. Finally, it is worth mentioning that drugs that did not use the *CYP2D6* pathway in their metabolism were predominantly prescribed at ^SUP^DDR, but the association was not significant.

### 2.5. Case Reports Series

**Figure 3:**
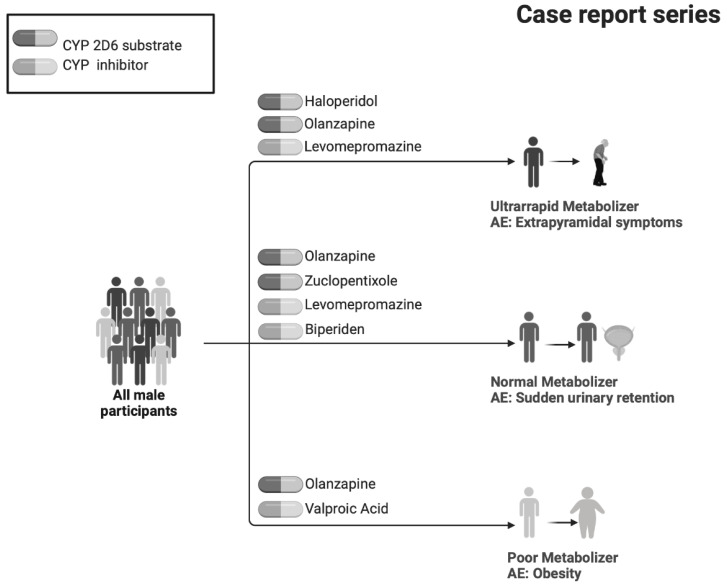
Participants’ cases that illustrate the importance of this study in a naturalistic environment.

All cases were male, aged 24–34 years old. CASE (A) was a UM participant with symptoms of extrapyramidal disorder (treated with haloperidol, olanzapine, and levomepromazine); all medications had an appropriate DDR. The UM phenotype quickly metabolizes haloperidol and olanzapine *CYP2D6* substrates, but levomepromazine is an inhibitor, so a rare AE according to the medications’ technical sheet appeared in this participant. CASE (B) was a NM with acute urinary retention (treated with zuclopentixol, biperidene, olanzapine, and levomepromazine). Levomepromazine and biperidene are strong *CYP2D6* inhibitors, and levomepromazine inhibits *CYP1A2*. Olanzapine is a substrate of *CYP2D6* and CYP1A2, and both enzymes had their function impaired due to the high number of inhibitors that this participant took simultaneously, so the urinary retention could be caused by these drug–drug interactions. Finally, CASE (C) was a PM subject with obesity (treated with valproic acid and olanzapine). This phenotype tends to accumulate medications that are substrates of enzymes’ metabolism, so the dose of olanzapine accumulated, causing the AE of weight gain, as described in its summary of product characteristics (see Figure 3).

## 3. Discussion

Our data evidenced that *CYP2D6* extreme phenotypes were linked to polypharmacy, different dose regimens, and tolerability. On one hand, *CYP2D6* UMs had the highest number of drug combinations and doses outside the recommended ones. On the other hand, *CYP2D6* PMs gathered higher rates of neurological and psychiatric AEs, being 33 times higher when a new antipsychotic drug was prescribed together with a *CYP2D6* inhibitor medication. These data highlight the need to carefully consider patient genotype in some scenarios when doses are exceeded without effective results. Furthermore, knowing patient phenotype can modify the benefit–risk balance when prescribing a new pharmacological treatment in ASD, especially antipsychotics, as their use is linked in this work to an increased risk of comorbidities or AEs at higher rates than described.

The results provide clear directions for clinical practice. Firstly, the long-term effectiveness of an outpatient pharmacovigilance program could allow the easy detection of safety problems in health systems. Secondly, it demonstrates the advantages of incorporating *CYP2D6* phenotype in a regular safety-monitoring system to understand interindividual differences in terms of tolerability and to fix existing dose ranges in each subject. Thirdly, it supports being aware of extreme *CYP2D6* phenotypes, mainly before starting several simultaneous prescriptions. In addition, the phenotypes can predict some unfavorable response patterns to certain treatments. All of this information can provide a better understanding of autism in a population under polypharmacy that, together with other clinical variables, may give us the key to constructing a safety risk prediction model.

One of the strengths is that our results provide real-world information about patients with ASD. These were 83% middle-aged men under multidrug treatment with a median of three identified AEs in autism. Surprisingly, intellectual disability, overweight condition, and sleep disorders were underdiagnosed. Multiple AEs associated with antipsychotics have been established in adults, including weight gain, sedation, prolongation of corrected QT interval, tardive and withdrawal dyskinesia, diabetes mellitus, and hyperlipidemia [23]. ASD subjects are at higher risk than regular adults for AEs following antipsychotic exposure, and emerging evidence indicates that they are at higher risk for weight gain and metabolic abnormalities compared with adults [24].

In this regard, *CYP2D6* PM participants were more likely to have drug doses outside the regular range and more comorbidities related to nervous or psychiatric disorders, whilst UMs were less likely to have ^SUP^DDR. Thus, *CYP2D6*-metabolizing phenotype, if know beforehand, could have an influence on prescription dose, being a future pharmacogenomic marker for this population. Previously, *CYP2D6* has been studied to evaluate its role in the development of AEs considering the different polymorphisms of the enzyme [25,26], as well as some drug-resistant adverse events [13], but all studies have been limited by low sample size. In this study, the contingency analysis showed that prescriptions at DDR doses or ^SUP^DDR were positively related with drugs not metabolized by cytochrome and cytochrome substrates. However, the presence of an inhibitory drug was negatively related, which is why there was a trend toward prescriptions at ^INF^DDR. Our results are in line with a study reporting the interaction between metoprolol and SRSI. They found that Paroxetine/fluoxetine initiation in metoprolol prescriptions, especially for older female patients, was associated with the risk of early discontinuation of metoprolol [27]. On the other hand, the IM, NM, and UM profiles were positively related with DDR, and the former with prescriptions at ^SUP^DDR. It would be logical to conclude that, for a UM profile, the prescription would be at ^SUP^DDR; however, due to the low sample size, a single UM patient was selected for the analysis who had all prescriptions at the DDR, so no conclusive results were possible in this part of the study.

Given that risperidone, together with aripiprazole are the only two molecules approved by Drug Regulatory Agencies for the treatment of ASD symptoms [28,29], increasing risperidone pharmacogenomics of *CYP2D6* knowledge is the key to personalized medication in terms of dose recommendation in ASD [30]. Since the effects of many factors in risperidone treatment are still being investigated, we believe this study makes a preliminary contribution toward personalized therapy considering pharmacogenomics when using risperidone and other drugs in autism. Further research is needed to avoid combinations of drugs formed by substrates and inhibitors of the same metabolic pathway.

In addition, many drugs used chronically in psychiatric diseases exert their effects mainly through other receptors, such as dopamine D2, which can have a mutual influence [31]. Hence, genetic screening for *CYP2D6* polymorphisms could help to predict unexpected adverse events caused by the higher plasma concentration of risperidone [30]. Together with pharmacogenomic implementation in patients with ASD, providers should work on methods to improve the distinction between comorbidities or AEs caused by medications in patients with ASD. Other markers, such as *CYP2C19* and *CYP3A4*, would also be suitable candidates to genotype in future studies; however, CYP2D6 was chosen since risperidone and aripiprazole are both substrates of this enzyme [28,29].

Some limitations should be under consideration. The sample was calculated using a convenience sample of ASD subjects, but the total number of subjects studied was relatively small. Working with a group with ASD and ID is an obvious challenge due to their low cooperation in a research environment, and their rigid routines represent a challenge, as introducing any change in their agenda (such as blood sample extraction) requires a great deal of coordination. This can compromise the statistical power to find differences and conditioned valid statistical conclusions, all the more so when related with genetics simple alleles, duplications, and deletions, amongst other calculations. In line with this, the authors agree that, for the final part of the study corresponding to the evaluation of the metabolizing profile of each patient, a multinomial logistic regression would enrich the results [32]. However, even if we used this analysis as guidance for contingency tables, due to the data distribution this option was finally discarded as it did not guarantee sufficient statistical power. Sample collection by saliva using cotton swabs was ruled out due to attempts by patients to swallow them in previous studies. Furthermore, the population included came from a local center in Alicante (Spain), so it may not represent other more diverse populations. In addition, pharmacological data were obtained from EHRs, and potential mismatches between patients’ intakes and prescribed doses could exist. Also, AEs were self-declared by health professionals without using any validated instrument. This may threaten future reproducibility and, therefore, external validity.

## 4. Method

### 4.1. Study Design and Ethics

An observational ambispective study was conducted over 36 months from January 2015 to January 2018, with 24 months of retrospective revision of participants’ health records and 12 months of prospective study. All adults with ASD were inhabitants of a residential facility (Infanta Leonor, San Rafael, APNAV, or EDUCATEA) and attended the Alicante Health Department—General Hospital (Alicante, Spain). The Ethics Committee board of Alicante Department of Health-General Hospital approved the protocol and all procedures of this study and UCAM University (Ethic Committee code 2016/02 and CE022211, respectively). This study was carried out in accordance with the requirements expressed in the Declaration of Helsinki, the guidelines of good practices of the ICH, and the current legislation in Spain in accordance with the provisions of ministerial order SAS/3470/2009 relative to the performance of observational studies. Further details of the local pharmacovigilance system created were previously reported in other work [2].

### 4.2. Participants

The sample size was calculated considering the available 250 participants with ASD who attended hospital consults. With a confidence level of 95% and a margin error of 10%, the minimum sample needed was 70 participants. Subjects were over 18 years old and had a diagnosis of: (a) autism spectrum disorder supported by the Diagnostic and Statistical Manual of Mental Disorders Fifth Edition (DSM-IV or DSM-5) criteria and confirmed by a second clinician from the research team; and (b) intellectual disability (IQ score < 70) according to Spanish social services records. Participants and/or legal guardians received and understood the study information, design, and purposes and signed consent forms.

### 4.3. Procedure

Two pharmacists and four clinical pharmacologists from the Clinical Pharmacology Service and Institute of Sanitary and Biomedical Research of Alicante (ISABIAL, Alicante General Hospital, Alicante, Spain), comprised the research team. They contacted ASD centers and held meetings with the management team, who contacted legal representatives/family/social services to inform them about the objectives of the present study. Later, individual meetings with the researchers were arranged to give the appropriate information and obtain participants’ informed consent. Once it was signed, the electronic or paper medical records were reviewed to collect the study variables in collaboration with the healthcare team. Both teams reviewed any divergence between the records. The healthcare team and participants’ families were subsequently informed about the results of the study through individual and collective meetings.

### 4.4. Data Collection

Data were obtained from a pharmacovigilance study based on data collection of patient comorbidities and pharmacological treatment (number of ongoing medications of antipsychotics, antidepressants, anxiolytics, and anticonvulsants). The prescription dose was classified following the dose range recommended on the technical data sheet or the summary of product characteristics at the DDR (dose in regular range) or outside (inferior (^INF^DDR) or superior (^SUP^DDR) doses). Reports of AEs and suspected adverse drug reactions (ADRs) notified by physicians were registered, analyzing drug causality as per the regular reports. Notifications were based on national and international guidelines for submitting adverse events reports for publication. Participants’ electronic health record (EHR) data were obtained using the Valencian Integrated Health Information System for recording patient information (ABUCASIS) filled by practitioners.

### 4.5. Comorbidities and AEs

The definitions for the purpose of this study were as follows: (a) comorbidities were defined as medical conditions that existed at the time of diagnosis of the index disease; (b) AEs were defined as any untoward medical occurrence in a patient to whom a pharmaceutical product had been administered and did not necessarily need to have a causal relationship with this treatment. They were collected throughout the follow-up period and coded with MedDRA (Version 12.0; International Federation of Pharmaceutical Manufacturers and Associations, Geneva, Switzerland).

### 4.6. CYP2D6 Genotyping and Phenotyping

The genetic profile of each patient was derived from blood samples taken in routine blood testing or from an extra blood extraction performed after informed consent. Blood samples were stored at −80 degrees Celsius. Genomic DNA was extracted following the manufacturer’s instructions with a QIAamp DNA Mini Kit (QUIAGEN). XL-polymerase chain (XL-PCR) analysis was used for the identification of duplications and deletions. This analysis was based on the instructions of the Spanish Consortium of Pharmacogenetics (CEIBA) and the Pharmacogenomics Iberoamerican Network (RIBEF) for the analysis of samples. DNA concentrations were quantified from absorbance at 260 nm using a Nanodrop 1000 spectrophotometer (Thermo Scientific, Waltham, MA, USA). The CYP2D6 genotyping comprised different simple alleles (*2, *3, *4, *6, *10, *17, *29, *35, *41), gene duplications, the presence or absence of allele 5 (3.5 kb), and gene copy number variations. The phenotype classification resulted in the three main metabolizing profiles obtained by the activity score (AS) developed by Caudle and collaborators [33]. To calculate the AS, the alleles were grouped according to their functionality and were given a value: AS = 0 (* 3/* 4, * 4/* 4, * 5/* 5, * 5/* 6); AS = 0.25 (* 4/* 10), AS = 0.5 (* 4/* 41, * 10/* 10), AS = 0.75 (* 10/* 41); AS =1 (* 41/* 41, * 1/* 5); AS = 1.25 (* 1/* 10); AS = 1.5 (* 1/* 41, * 1/* 9); AS = 2 (* 1/* 1, * 1/* 2); AS = 2.25 (* 2 × 2/* 10); and AS > 2.25 (* 1/* 1 × N, * 1/* 2 × N^b^, * 2^a^/* 2 × N^b^, * 1 × 2/* 9).

The sum of the scores of both alleles defined the four phenotypes: poor metabolizer (PM, AS = 0), intermediate (IM, 0 < x < 1.25), normal (NM, 1.25 ≤ x ≤ 2.25), or ultrarapid (UM, >2.25). The alleles and their functionality varied strongly from one ethnic group to another [34]. For the Caucasian population, it was described that between 5–10% are PM and 1–2% are UM, the rest being IM or NM, and in the Mediterranean area, a slightly higher UM frequency was found between 7–10% [35]. This is why, for the evaluation of the metabolizing phenotype, the 10 most frequent alleles in all populations were genotyped (* 2, * 3, * 4, * 5, * 6, * 10, * 17, * 29, * 35, * 41), as well as their deletions and duplications and variations in the number of copies [33].

### 4.7. Drug Reports: CYP2D6 Phenotype Applied to Clinical Cases

Once the analysis of all the variables was carried out, CYP2D6 phenotype was calculated in every participant. For each case, knowing the metabolizing phenotype helped to understand the appearance rate of some AEs, as well as some medication doses. Here, we presented three of the most relevant clinical cases in terms of severity in AEs, each with a different metabolizing profile (PM, EM, and UM). The relationship between the drugs and the enzymes (inducer, inhibitor, substrate, or active metabolite), the metabolic activity of medications and their metabolites, and the drug combinations presented, were analyzed.

### 4.8. Statistical Analysis

Quantitative parametric data were presented as mean ± standard deviation (SD), while medians (interquartile ranges) were used for nonparametric data. Comparisons for continuous or categorical data between two groups were conducted using an independent *t*-test or chi-squared test (or Fischer exact test), respectively. Also, multiple logistic regressions were performed to investigate the relationships between biological sex, age, BMI, AEs, number of ongoing medications (antipsychotics, antidepressants, anxiolytics, and mood stabilizers), number of comorbidities, and CYP2D6 metabolizer phenotype. Observed gene frequencies were compared using the chi-squared (×2) goodness-of-fit test and the Hardy–Weinberg proportion. Due to the low number of homozygotes for each polymorphism, for analyses, patients were grouped as carriers or noncarriers, defined as participants who tested positive for the presence of the allelic variants (dominant model). The odds ratio (OR) was calculated using a logistic regression analysis before and after adjustment for other pharmacology factors, inserting the term interaction in the model. For interaction analyses, genotypes were also classified into dichotomous variables according to dominant models. A *p* ≤ 0.05 was considered statistically significant. In all cases, multiple testing was adjusted using Bonferroni correction. All statistical analyses were carried out using R (version 3.2.0). In the drug reports, an adaptation of the Cochran–Mantel–Haenszel test [36] was used to show the values obtained, stratified by site and using a color code to highlight the most extreme results. All these data management techniques and analyses were performed using Excel (version 19.0) (Microsoft, Redmon, Washington, USA) and a simple and affordable color code in tables and graphs. In the final part of the study, corresponding to the evaluation of the metabolizing profile of each patient, SPSS (version 25) was used to perform a contingency analysis that related pharmacological interactions with enzymes or medication dose with enzyme phenotype.

## 5. Conclusions

Our prospective data evidenced that knowing patients’ phenotypes, especially if they were UM or PM, before prescription may reduce medications or AEs. This test is pertinent to improving risk rates when prescribing a new pharmacological treatment in ASD patients, especially an antipsychotic, as their use was linked to an increased risk for new comorbidities, which are sometimes different from regular drug side-effects. Further studies considering these potential interactions, as well as subsequent monitoring, could help us to understand the interindividual variability in autistic real-world subjects.

## Figures and Tables

**Table 1:** Autism spectrum disorder (ASD) participants classified by *CYP2D6* phenotype (poor metabolizers (PMs), intermediate metabolizers (IMs), normal metabolizers (NMs), or ultrarapid metabolizers (UMs)).

Population	ASD (n = 83) *	ASD-*CYP2D6* (n = 71)	PM (n = 5)	IM (n = 31)	NM (n = 32)	UM (n = 3)
Age (mean (SD) years)	30 (10)	30 (10)	27 (9)	29 (10)	30 (10)	33(10)
Sex (% male)	86%	82%	80%	84%	81%	100%
Intellectual disability in HER ** (IQ < 70)	20%	20%	20%	22.5%	17%	33%

* Espadas et al. (2020); ** all participants had an IQ < 70 according to social services registries but, in EHRs, only 20% of participants specified this.

**Table 2:** Frequencies of the most prevalent comorbidities according to *CYP2D6* phenotype.

**Poor Metabolizers (n = 5, 7%)**	**Comorbidities (n = 222)**
Nervous System (10/16)	Anxiety (2/10)
	Depression (1/10)
	Epilepsy (3/10)
	Headache (1/10)
	Insomnia (1/10)
	IQ < 70 (1/10)
	Oligophrenia (1/10)
Musculoskeletal System (2/16)	Scoliosis (1/2)
Integumentary System (2/16)	Acne (1/2)
	Dermatitis (1/2)
Digestive System (1/16)	Dyspepsia (1/1)
Blood and Lymphatic Tissues (1/16)	Anemia (1/1)
**Intermediate Metabolizers (n = 31, 44%)**	
Nervous System (29/101)	Anxiety (2/29)
	Dementia (4/29)
	Epilepsy (6/29)
	Insomnia (4/29)
	IQ < 70 (7/29)
	Nervous Agitation (4/29)
Renal and Urinary System (12/101)	Urinary Incontinence (11/12)
Digestive System (11/101)	Constipation (3/11)
Respiratory, Thoracic, and Mediastinal System (9/101)	Bronchitis (3/9)
	Asthma (3/9)
**Normal Metabolizers (n = 32, 45%)**	
Nervous System (30/96)	Anxiety (2/30)
	Epilepsy (4/30)
	Insomnia (5/30)
	IQ < 70 (5/30)
	Nervous Agitation (8/30)
Integumentary System (13/96)	Dermatitis (7/13)
Renal and Urinary System (11/96)	Urinary Incontinence (10/11)
Digestive System (9/96)	Constipation (7/9)
**Ultra-Rapid Metabolizers (n = 3; 4%)**	
Nervous System (3/9)	Insomnia (2/3)
	IQ < 70 (1/3)
Renal and Urinary System (1/9)	Urinary Incontinence (1/1)
Digestive System (1/9)	Malocclusion (1/1)
Respiratory System (2/9)	Asthma (1/2)
	Nasal Polyps (1/2)
Vascular System (1/9)	Subclavian Artery Compression Syndrome (1/1)
Genetic Diseases (1/9)	Fragile X Syndrome (1/1)

**Table 3:** Description of medications taken simultaneously according to participants’ phenotypes.

Poor Metabolizers—Median (IQR) 4 (3–5) Drugs/Patient				
**Drug**	Median (IQR)	Prescribed Dosage		Main Drugs Prescribed
Antipsychotic	2 (1–3)/patient	DDR:	100%	Quetiapine (29%, *CYP3A4* substate)
				Risperidone (21%, *CYP2D6* substate)
				Levomepromazine (14%, *CYP2D6* inhibitor)
Antidepressant	1 (0.5–1)/patient	^SUP^DDR:	17%	Sertraline (33%, *CYP2D6* inhibitor)
		DDR:	67%	Fluoxetine (17%, *CYP2D6* inhibitor)
		^INF^DDR:	17%	Fluvoxamine (17%, *CYP2D6* substate)
Anticonvulsant	1 (0–2)/patient	DDR:	100%	Lamotrigine (40%, NA)
				Valproic Acid (20%, *2C19* substrate)
				Levetiracetam (20%, *2C19* substrate)
Anxiolytic	1 (0–2)/patient	DDR:	100%	Lorazepam (40%, NA)
				Lormetazepam (40%, NA)
				Clonazepam (20%, NA)
**Intermediate Metabolizers—Median (IQR) 4 (2–7) Drugs/Patient**				
**Drug**	**Median (IQR)**	**Prescribed Dosage**		**Main Drugs Prescribed**
Antipsychotic	1 (1–2)/patient	^SUP^DDR:	7%	Risperidone (19%, *CYP2D6* substate)
		DDR:	93%	Levomepromazine (14%, *CYP2D6* inhibitor)
		^INF^DDR:	0%	Olanzapine (12%, *CYP1A2* substrate)
Antidepressant	0 (0–1)/patient	DDR:	100%	Fluvoxamine (63%, *CYP2D6* substate)
				Fluoxetine (25%, *CYP2D6* inhibitor)
				Trazodone (13%, *CYP3A4* substrate)
Anticonvulsant	1 (0–2)/patient	^SUP^DDR:	15%	Topiramate (30%, *CYP2C19* inhibitor)
		DDR:	85%	Valproic Acid (18%, *CYP2C19* substrate)
		^INF^DDR:	0%	Carbamazepine (12%, *CYP2C19* inducer)
Anxiolytic	0 (0–1)/patient	^SUP^DDR:	9%	Clorazepate (27%, NA)Clonazepam (23%, NA)Lormetazepam (18%, NA)
		DDR:	86%	
		^INF^DDR	5%	
**Normal Metabolizers—Median (IQR) 4 (2–6) Drugs/Patient**				
**Drug**	**Median (IQR)**	**Prescribed Dosage**		**Main Drugs Prescribed**
Antipsychotic	1 (1–3)/patient	^SUP^DDR:	11%	Risperidone (16%, *CYP2D6* substate)
		DDR:	88%	Levomepromazine (16%, *CYP2D6* inhibitor)
		^INF^DDR:	1%	Olanzapine (16%, *CYP1A2* substrate)
Antidepressant	0 (0–1)/patient	^SUP^DDR	0%	Fluvoxamine (35%, *CYP2D6* substate)
		DDR:	96%	Sertraline (17%, *CYP2D6* inhibitor)
		^INF^DDR:	4%	Trazodone (13%, *CYP3A4* substrate)
Anticonvulsant	1 (0–1)/patient	^SUP^DDR	6%	Topiramate (28%, *CYP2C19* inhibitor)
		DDR:	94%	Valproic Acid (22%, *CYP2C19* substrate)
		^INF^DDR:	0%	Oxcarbazepine (13%, *CYP3A4* inducer)
Anxiolytic	0 (0–1)/patient	^SUP^DDR	14%	Clonazepam (29%, NA)
		DDR:	86%	Lormetazepam (19%, NA)
		^INF^DDR R:	0%	Diazepam (19%, *CYP2C19* inducer)
**Ultra-Rapid Metabolizers—Median (IQR) 6 (3–6) Drugs/Patient**				
Antipsychotic	3 (1–4)/patient	DDR:	100%	Haloperidol (22%, *CYP2D6* substrate)
				Olanzapine (22%, *CYP1A2* substrate)
				Amisulpride/Levomepromazine/
				Quetiapine/
				(11%, *CYP2D6* inhibitor)
Antidepressant	0 (0–1)/patient	DDR:	100%	Fluoxetine (100%, *CYP2D6* inhibitor)
Anticonvulsant	0 (0–1)/patient	DDR:	100%	Topiramate (100%, *CYP2C19* inhibitor)
Anxiolytic	No reported use in UMs		-	-

DDR: daily dose recommended; IQR: interquartile range; NA: not available. All cytochrome references were obtained from pharmgkb.org.

**Table 4:** Association between dosage, drug metabolism, and phenotype.

	^INF^DDR	DDR	^SUP^DDR	*p*-Value
**Metabolizer phenotype**				0.000035
PM	100%	0%	0%	
EM (IM, NM)	0%	84.2%	100%	
UM	0%	15.8%	0%	
**Drug metabolism**				0.683
*CYP2D6* substrate	50%	57.9%	75%	
*CYPD26* inhibitor	50%	27.3%	0%	
No interaction	0%	15.8%	25%	

## Data Availability

Data will be accessible upon request.
